# Investigating the role of heat shock protein 47 in fibrosis in Crohn’s disease

**DOI:** 10.1038/s41598-022-15153-2

**Published:** 2022-06-29

**Authors:** Hiroki Kurumi, Tomoaki Takata, Tsutomu Kanda, Takaaki Sugihara, Tomoyuki Kakugawa, Shin-ichi Yokota, Tomohito Morisaki, Taro Akashi, Hajime Isomoto

**Affiliations:** 1 Division of Gastroenterology and Nephrology, Tottori University Faculty of Medicine, 36-1, Nishi-cho, Yonago, Tottori 683-8504 Japan; 2 Department of Pulmonology and Gerontology, Graduate School of Medicine, Yamaguchi University, 1-1-1, Minamikogushi, Ube, Yamaguchi 755-8505 Japan; 3 Department of Microbiology, Sapporo Medical University School of Medicine, Minami-1-jo-Nishi-17, Chuo-ku, Sapporo, Hokkaido 060-8556 Japan; 4 Department of Endoscopy, Nagasaki University Hospital, 1-7-1, Sakamoto, Nagasaki, 852-8501 Japan

**Keywords:** Biomarkers, Gastroenterology, Medical research

## Abstract

Crohn’s disease (CD) and ulcerative colitis (UC) are chronic inflammatory disorders of the gastrointestinal tract that share similar genetic risk factors. However, while fibrotic stricture of the intestine is a major characteristic of CD; it is rarely observed in UC. Deposition of collagen in the extracellular matrix contributes to the formation of fibrotic strictures in CD, but the underlying mechanisms are unknown. In the present study, we found that heat shock protein 47 (HSP47), a stress-response protein that acts as a molecular chaperone during the processing and secretion of collagen, expressed in the intestinal tissue from patients with CD. Serum HSP47 levels and anti-HSP47 antibody titers were significantly higher in patients with CD than in those with UC. Furthermore, anti-HSP47 antibody levels correlated significantly with fibrosis in CD. In addition, HSP47 inhibition significantly suppressed collagen production in fibroblasts in vitro. These findings suggest that HSP47 is a biomarker for differentiating fibrotic from non-fibrotic forms of CD. Additionally, we propose that HSP47 could be a potential target for treating fibrosis in patients with CD.

## Introduction

Inflammatory bowel disease (IBD) is a chronic inflammatory disorder of the gastrointestinal tract characterized by an inappropriate inflammatory response to intestinal microbes^1^. IBD is typically further classified as ulcerative colitis (UC) or Crohn’s disease (CD)^2^. Recent investigations have identified a cluster of genetic susceptibility loci for IBD^3,4^. Both UC and CD often occur within the same family, suggesting that the relevant genes are common to both diseases^1,5^. However, the clinical manifestations of these diseases differ. UC is limited to the colon and is characterized by superficial mucosal inflammation, sometimes leading to bleeding and toxic megacolon. In contrast, CD affects any part of the gastrointestinal tract and is characterized by transmural inflammation of discontinuous lesions, often leading to fistula and abscess formation^6^. Fibrotic strictures are a major complication of CD but are rarely observed in UC^7^. Deposition of collagen in the extracellular matrix contributes to fibrotic stricture formation at wound-healing sites^8^. Knowledge of the molecular mechanisms underlying intestinal fibrosis has enabled the identification of several anti-fibrotic therapeutic targets^9^; however, further investigation into the mechanism for fibrotic stricture formation in CD is required to establish novel therapeutic targets.

Heat shock proteins (HSPs) are ubiquitous proteins that act as molecular chaperones and regulate the biosynthesis, folding, transport, and assembly of cellular proteins^10^. HSPs are induced in response to various stresses. Among the several subtypes of HSPs, HSP47 is a collagen-specific chaperone expressed in the endoplasmic reticulum (ER), and it plays an essential role in the processing and secretion of collagen from the ER and its subsequent transport to the Golgi apparatus^11^. HSP47 levels correlated positively with the degree of fibrosis and the amount of collagen secreted in rats with induced liver cirrhosis^12^. In addition, the number of HSP47-positive pneumocytes was significantly higher in patients with idiopathic pulmonary fibrosis than in patients with bronchiolitis obliterans^13^. HSP47 is involved in fibrotic stricture formation in CD, but its role in this process is unknown.

Based on the above observations, we hypothesized that high HSP47 levels are a potential indicator of CD in patients with IBD and that it may reflect disease progression of CD. In this study, we aimed to investigate the role of HSP47 in patients with IBD, particularly in those with CD.

## Results

### Patient characteristics

A total of 22 healthy controls, 26 patients with UC, and 32 patients with CD were included in the analysis, and their main characteristics are presented in Table 1. Age, height, weight, and body mass index (BMI) differed significantly among the three groups. There was no difference in disease evolution time between the UC and CD groups. A CAI > 4 was observed in 10 patients (38.5%) and a CDAI > 150 was observed in 9 patients (28.1%). No difference was observed in the ratio of active stage to remission stage between UC and CD groups. Eighteen patients (56.3%) with CD had been diagnosed with stricturing. Regarding the treatments being administered to patients at the time of evaluation of serum HSP47 and anti-HSP47 antibody levels, we observed that steroids were used significantly more frequently for patients in the UC group, while infliximab was used significantly more frequently for patients in the CD group (P = 0.045 and P < 0.001, respectively).

**Table 1:** Patient characteristics.

Characteristics	Controls (n = 22)	UC (n = 26)	CD (n = 32)	*P*-value
Age, years	30.1 ± 8.2	43.0 ± 13.2	33.9 ± 11.7	0.015^a^
Sex, male/female	17/5	15/11	23/9	0.306^b^
Height, m	1.68 ± 0.09	1.62 ± 0.08	1.67 ± 0.06	0.023^a^
Weight, kg	63.6 ± 11.0	59.2 ± 16.0	55.4 ± 8.7	0.023^a^
BMI, kg/m^2^	22.2 (20.3–24.3)	21.2 (19.6–23.2)	19.1 (18.3–21.0)	< 0.01^c^
Clinical stage, active/remission		10/16	9/23	0.404^b^
Evolution time of disease, years		8.2 ± 7.0	12.1 ± 9.9	0.092^d^
**Montreal classification**				
Age of diagnosis, A1/A2/A3		6/26/0		
Location, L1/L1 + 4/L2/L2 + 4/L3/L3 + 4/L4		2/5/2/0/6/17/0		
Behaviour, B1 (B1p)/B2 (B2p)/B3 (B3p)/B2 + B3 ((B2 + B3) + p)		11 (5)/11(8)/3(3)/7(6)		
HSP47, pg/mL	195.2 (169.4–224.7)	158.8 (133.9–209.5)	245.6 (182.8–293.5)	< 0.01^c^
Anti-HSP47 antibody	0.15 ± 0.05	0.15 ± 0.07	0.35 ± 0.17	< 0.01^a^
**CAI, n (%)**				
≥ 6		9 (34.6)		
6 <		17 (65.4)		
**CDAI, n (%)**				
≥ 150			9 (28.1)	
< 150			23 (71.9)	
**Current treatment**				
5-ASA, n (%)		17 (65.4)	25 (78.1)	0.280^b^
Steroid, n (%)		9 (34.6)	4 (12.5)	0.045^b^
Azathioprine, n (%)		7 (26.9)	6 (18.8)	0.458^b^
Infliximab, n (%)		0 (0)	12 (37.5)	< 0.01^b^
GMA, n (%)		2 (7.7)	1 (3.1)	0.435^b^

Clinical active stage was defined as CAI > 4 for UC and CDAI > 150 for CD. Montreal classification of CD: A1, age < 16 years; A2, age 17–40 years; A3, age > 40 years. *L1* ileal, *L2* colonic, *L3* ileocolonic, *L4* isolated upper disease, *B1* non-structing and non-penetrating, *B2* structing, *B3* penetrating, *p* perianal, *UC* ulcerative colitis, *CD* Crohn’s disease, *BMI* body mass index, *HSP* heat shock protein, *CAI* clinical activity index, *CDAI* Crohn’s disease activity index, *GMA* granulocyte–monocyte apheresis. ^a^One-way analysis of variance; ^b^Chi-square test; ^c^Kruskal–Wallis test; ^d^Student’s *t* test.

### Differences in serum HSP47 and anti-HSP47 antibody levels among the control, UC, and CD groups

**Figure 1:**
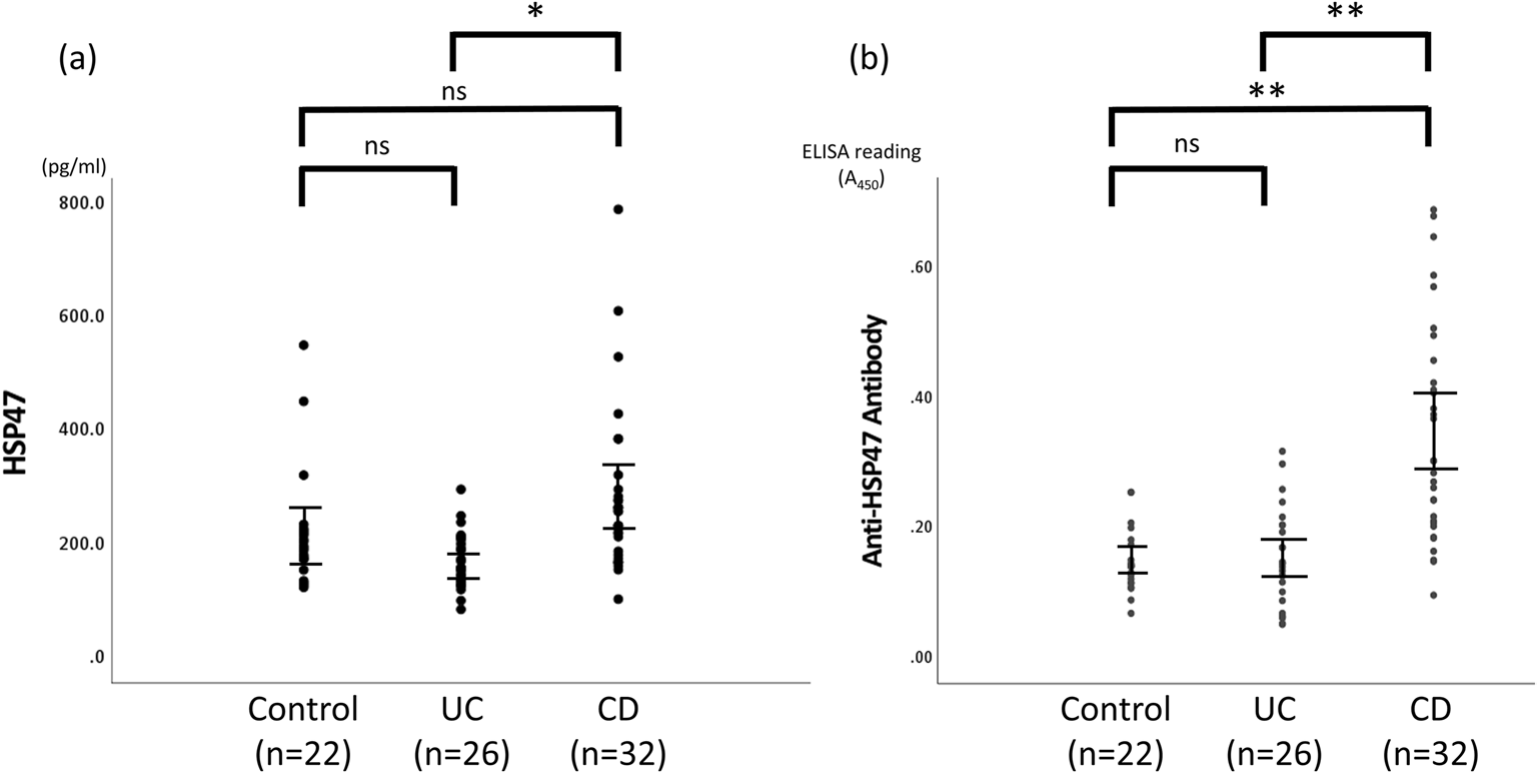
Comparison of serum HSP47 and anti-HSP47 antibody levels among control, UC, and CD groups. (**a**) Quantitative analysis of HSP47 levels in control (n = 22), UC (n = 26), and CD (n = 32) groups. (**b**) Quantitative analysis of anti-HSP47 antibody levels in control, UC, and CD groups. Each dot represents one subject, and bars indicate average ± SEM. *P < 0.001 (Dann–Bonferroni); **P < 0.001 (Scheffe test). *HSP* heat shock protein, *UC* ulcerative colitis, *CD* Crohn’s disease, *ns* not significant.

We compared serum HSP47 and anti-HSP47 antibody levels between the control, UC, and CD groups (Table 1). HSP47 levels were significantly higher in the CD group than in the UC group (P < 0.001). By contrast, neither the CD group and control group, nor the UC group and control group, differed significantly from each other (Fig. 1a). The CD group showed a significantly higher serum anti-HSP47 antibody titer than the UC and the control groups (P < 0.001, respectively), although the UC group and control group did not differ significantly from each other (Fig. 1b).

### Differences in serum HSP47 and anti-HSP47 antibody levels according to disease activity in CD

**Figure 2:**
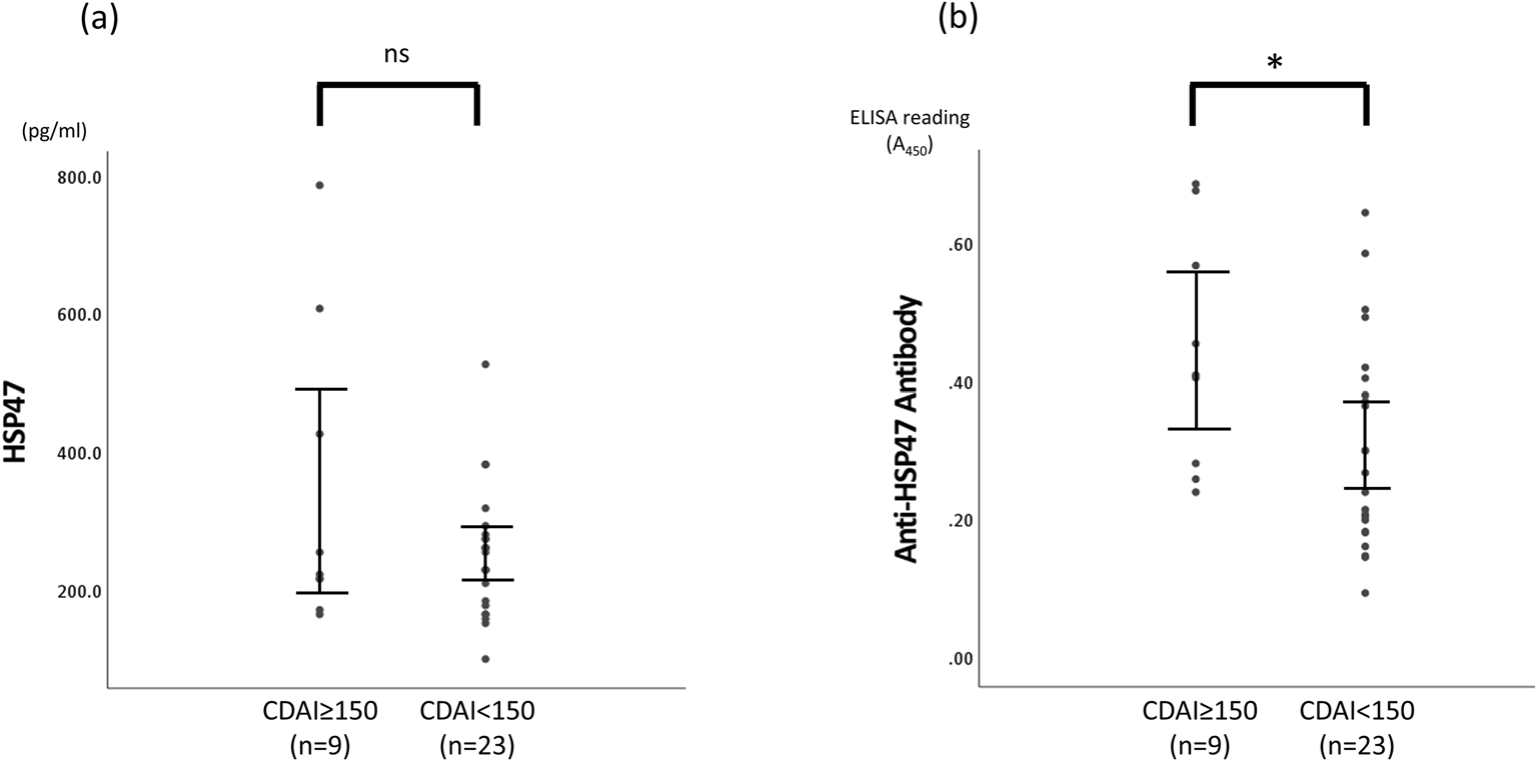
Comparison of serum HSP47 and anti-HSP47 antibody levels between CDAI < 150 and CDAI ≥ 150 groups. (**a**) Quantitative analysis of HSP47 levels in the CDAI ≥ 150 (n = 9) and CDAI (n = 23) groups. (**b**) Quantitative analysis of anti-HSP47 antibody levels in the CDAI < 150 and CDAI ≥ 150 groups. Each dot represents one subject, and bars indicate average ± SEM. **P < 0.001 (Student’s *t* test). *HSP* heat shock protein, *CDAI* Crohn’s disease activity index, *ns* not significant.

To compare serum HSP47 and anti-HSP47 antibody levels according to the disease activity of CD, patients with CD were classified into two groups: a clinically active stage defined as CDAI ≥ 150 and a clinically remission stage defined as CDAI < 150 (Table 2). The CDAI ≥ 150 and CDAI < 150 groups did not differ significantly from each other in age, sex, height, weight, BMI, or current treatment. HSP47 levels did not vary significantly with CD activity (P = 0.77) (Fig. 2a). By contrast, serum anti-HSP47 antibody levels were significantly higher in the CDAI ≥ 150 group than in the CDAI < 150 group (P = 0.033) (Fig. 2b).

**Table 2:** Characteristics of patients with CD by disease activity.

Characteristics	CDAI ≥ 150 (n = 9)	CDAI < 150 (n = 23)	*P*-value
Age, years	32.0 (21.5–38.5)	33.0 (24.0–47.0)	0.564^a^
Sex, male/female	5/4	18/5	0.199^b^
Height, m	1.65 ± 0.06	1.67 ± 0.06	0.251^c^
Weight, kg	51.3 ± 2.7	56.9 ± 9.7	0.099^c^
BMI, kg/m^2^	18.8 ± 1.6	20.0 ± 2.4	0.175^c^
Evolution time of disease, years	12.6 ± 11.3	11.9 ± 9.5	0.872^c^
**Montreal classification**			
Age of diagnosis, A1/A2/A3	3/6/0	3/20/0	0.186^b^
Location, L1/L1 + 4/L2/L2 + 4/L3/L3 + 4/L4	0/1/1/0/1/6/0	2/4/1/0/5/11/0	0.691^b^
Behaviour, B1 (B1p)/B2 (B2p)/B3 (B3p)/B2 + B3 ((B2 + B3) + p)	2(2)/3(2)/1(1)/3(2)	9 (5)/8(6)/2(2)/4(4)	0.571^b^
HSP47, pg/mL	226.3 (197.4–520.3)	258.4 (181.2–283.9)	0.771^a^
Anti-HSP47 antibody	0.45 ± 0.17	0.31 ± 0.15	0.033^c^
**Current treatment**			
5-ASA, n (%)	2 (22.2)	7 (30.4)	0.642^b^
Steroid, n (%)	0 (0)	3 (13.0)	0.255^b^
Azathioprine, n (%)	2 (22.2)	4 (17.4)	0.753^b^
Infliximab, n (%)	4 (44.4)	8 (34.8)	0.612^b^
GMA, n (%)	1 (11.1)	0 (0)	0.104^b^

Montreal classification of CD: A1, age < 16 years; A2, age 17–40 years, A3, age > 40 years. *L1* ileal, *L2* colonic, *L3* ileocolonic, *L4* isolated upper disease, *B1* non-structing and non-penetrating, *B2* structing, *B3* penetrating, *p* perianal, *CDAI* Crohn’s disease activity index, *BMI* body mass index, *HSP* heat shock protein, *GMA* granulocyte–monocyte apheresis. ^a^Mann–Whitney *U* test; ^b^Chi-square test; ^c^Student’s *t* test.

### Differences in serum HSP47 and anti-HSP47 antibody levels between patients with CD with and without intestinal stricturing

**Figure 3:**
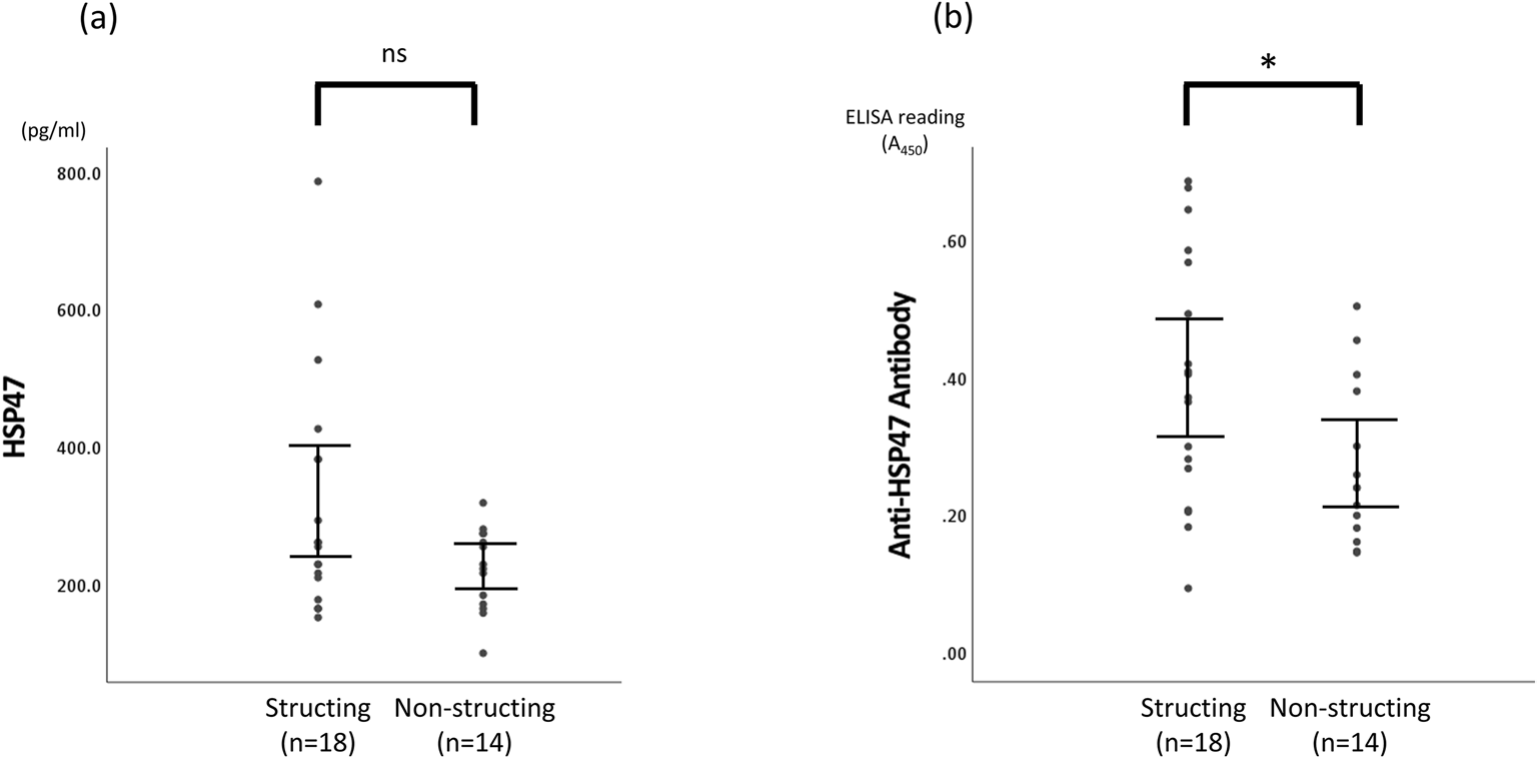
Comparison of serum HSP47 and anti-HSP47 antibody levels between stricturing and nonstricturing disease in patients with CD. Quantitative analysis of (**a**) HSP47 and (**b**) anti-HSP47 antibody levels in stricturing and nonstricturing groups in patients with CD. (**b**) Quantitative analysis of anti-HSP47 antibody in CD patients with and without intestinal stricturing. Each dot represents one subject, and bars indicate average ± SEM. *P = 0.033 (Student’s *t* test). *HSP* heat shock protein, *CDAI* Crohn’s disease activity index, *ns* not significant.

To compare serum HSP47 and anti-HSP47 antibody levels between patients with CD with and without intestinal stricturing, the patients were classified into two sub-groups: stricturing and nonstricturing groups (Table 3). The stricturing and nonstricturing groups had similar patient characteristics, including age, sex, height, weight, clinical stage, evolution time of disease, and location of disease. Serum HSP47 levels were not significantly different between the stricturing group and nonstricturing group (P = 0.21) (Fig. 3a), but serum anti-HSP47 antibody levels were significantly higher in the stricturing group than in the nonstricturing group (P = 0.03) (Fig. 3b).

**Table 3:** Characteristics of patients with CD with and without intestinal structuring.

Characteristics	Stricturing (n = 18)	Non-stricturing (n = 14)	*P*-value
Age, years	34.4 ± 12.2	33.3 ± 11.4	0.786^a^
Sex, male/female	14/4	9/5	0.400^b^
Height, m	1.69 (1.62–1.70)	1.69 (1.64–1.74)	0.587^c^
Weight, kg	52.5 ± 4.7	59.0 ± 11.2	0.056^a^
BMI, kg/m^2^	18.5 (18.2–20.5)	20.3 (18.7–22.1)	0.022^c^
Clinical stage, active/remission	6/12	3/11	0.683^b^
Evolution time of disease, years	13.2 ± 10.1	10.6 ± 9.7	0.472^a^
**Montreal classification**			
Age of diagnosis, A1/A2/A3	6/12/0	0/14/0	0.017^b^
Location, L1/L1 + 4/L2/L2 + 4/L3/L3 + 4/L4	1/3/0/0/2/12/0	1/2/2/0/4/5/0	0.255^b^
HSP47, pg/mL	261.6 (205.4–396.6)	229.5 (173.2–277.5)	0.206^c^
Anti-HSP47 antibody	0.40 ± 0.18	0.28 ± 0.12	0.033^a^
**Current treatment**			
5-ASA, n (%)	3 (16.7)	6 (42.9)	0.132^b^
Steroid, n (%)	2 (11.1)	2 (14.3)	0.788^b^
Azathioprine, n (%)	1 (5.6)	5 (35.7)	0.064^b^
Infliximab, n (%)	8 (44.4)	4 (28.6)	0.471^b^
GMA, n (%)	1 (5.6)	0 (0)	0.370^b^

Clinical active stage was defined as CAI > 4 for UC and CDAI > 150 for CD. Montreal classification of CD: A1, age < 16 years; A2, age 17–40 years, A3, age > 40 years, *L1* ileal, *L2* colonic, *L3* ileocolonic, *L4* isolated upper disease, *B1* non-structing and non-penetrating, *B2* structing, *B3* penetrating, *p* perianal, *CDAI* Crohnʼs disease activity index, *BMI* body mass index, *HSP* heat shock protein, *GMA* granulocyte-monocyte apheresis. ^a^Student’s *t* test; ^b^Chi-square test; ^c^Mann–Whitney *U* test.

### Optimal cut-off values for serum HSP47 and anti-HSP47 antibody levels for differentiating between CD and UC

**Figure 4:**
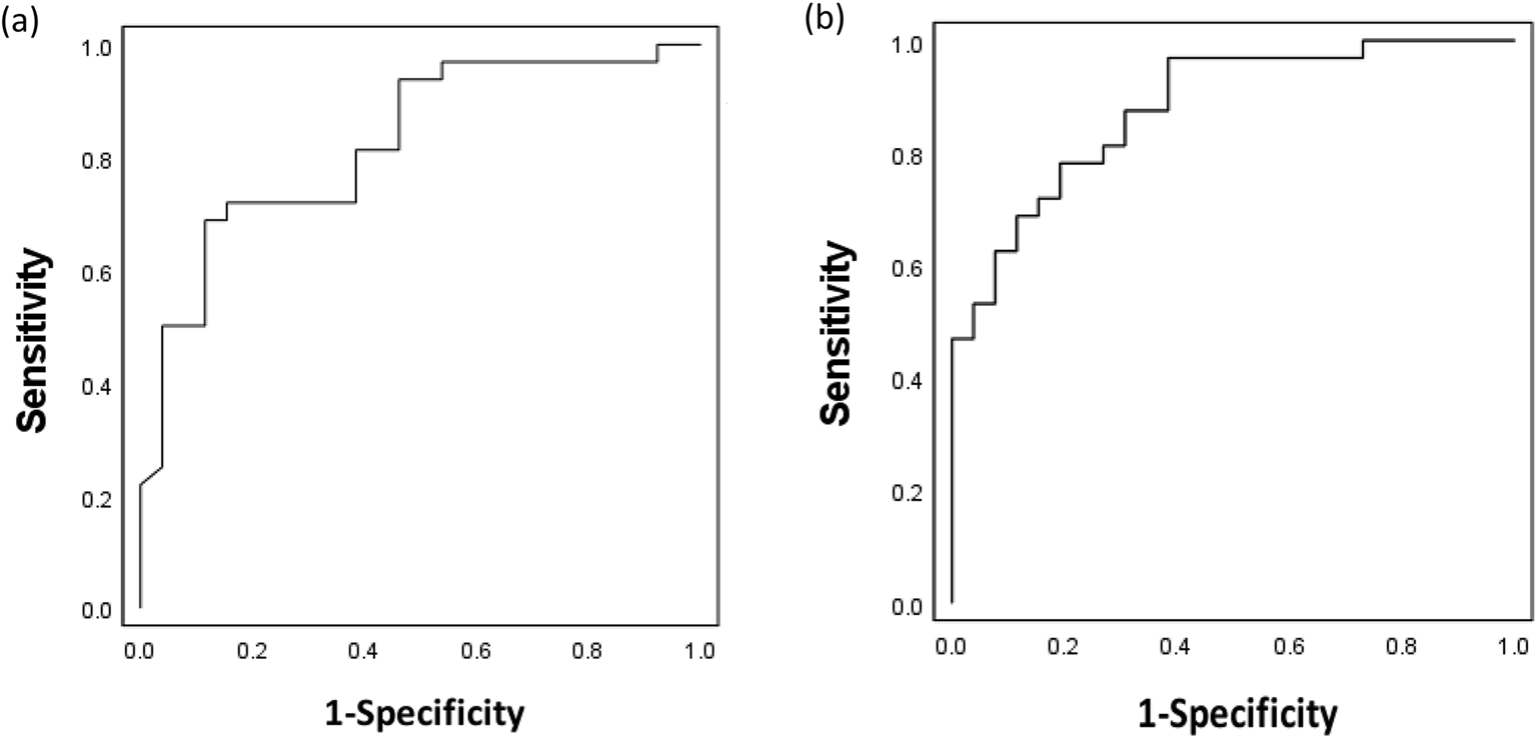
ROC curves of HSP47 and anti-HSP47 antibody for differentiating CD from UC. (**a**) ROC curve of HSP47 showing an AUC of 0.824. (**b**) ROC curve of anti-HSP47 antibody showing an AUC of 0.880. *HSP* heat shock protein, *CD* Crohn’s disease, *UC* ulcerative colitis, *ROC* receiver operating characteristics, *AUC* area under the curve.

Receiver operating characteristic (ROC) curve analyses were performed to define the cut-off values of serum HSP47 and anti-HSP47 antibody levels for differentiating between CD and UC. The area under the curve (AUC) was 0.824 for serum HSP47 levels (Fig. 4a) and 0.880 for serum anti-HSP47 antibody levels (Fig. 4b), indicating that both measurements can be used to accurately differentiate CD from UC. The ROC curve analyses determined a cut-off value of 212.9 pg/mL for serum HSP47 levels, with a sensitivity of 71.8% and a specificity of 84.6%, and a cut-off value of 0.207 (A_450_) for serum anti-HSP47 antibody levels, with a sensitivity of 78.1% and a specificity of 80.7%.

### Optimal cut-off values for serum anti-HSP47 antibody levels for differentiating CD with or without intestinal stricturing

**Figure 5:**
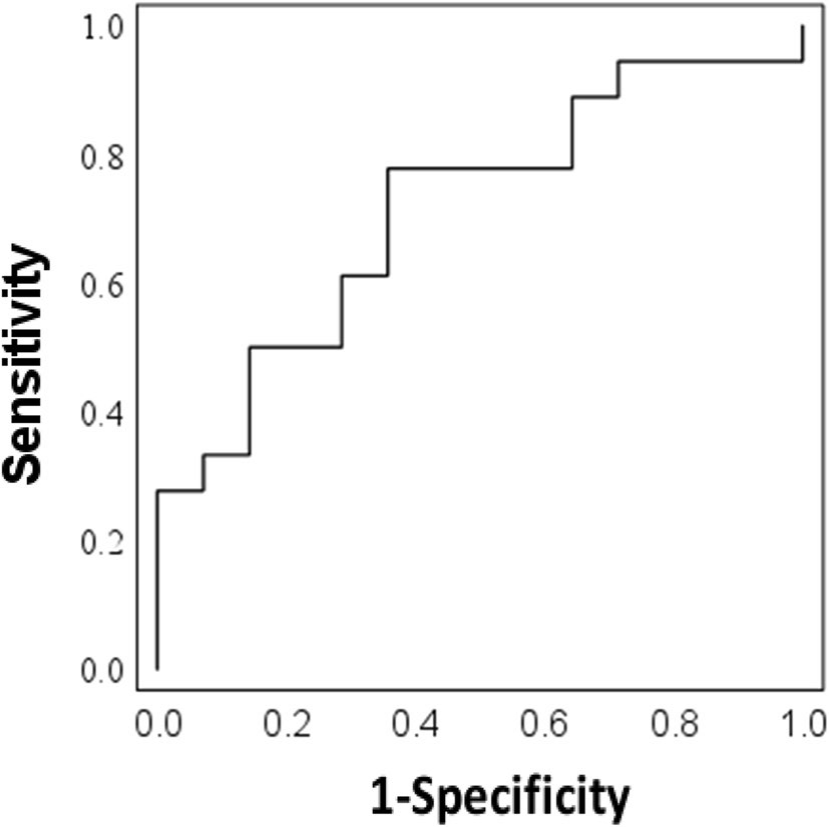
ROC curves of anti-HSP47 antibody for differentiating CD with or without intestinal stricturing. ROC curve of anti-HSP47 antibody showing an AUC of 0.714. *HSP* heat shock protein, *CD* Crohn’s disease, *UC* ulcerative colitis, *ROC* receiver operating characteristics, *AUC* area under the curve.

ROC curve analyses were performed to define the cut-off values of serum anti-HSP47 antibody levels for differentiating CD with or without stricturing. The AUC was 0.714 (Fig. 5), indicating that the measurement can be used to accurately differentiate between CD with or without intestinal stricturing. The ROC curve analyses determined a cut-off value of 0.267 (A_450_) for serum anti-HSP47 antibody levels, with a sensitivity of 77.8% and a specificity of 64.3%.

### Expression of HSP47 in the colonic mucosa of patients with CD

**Figure 6:**
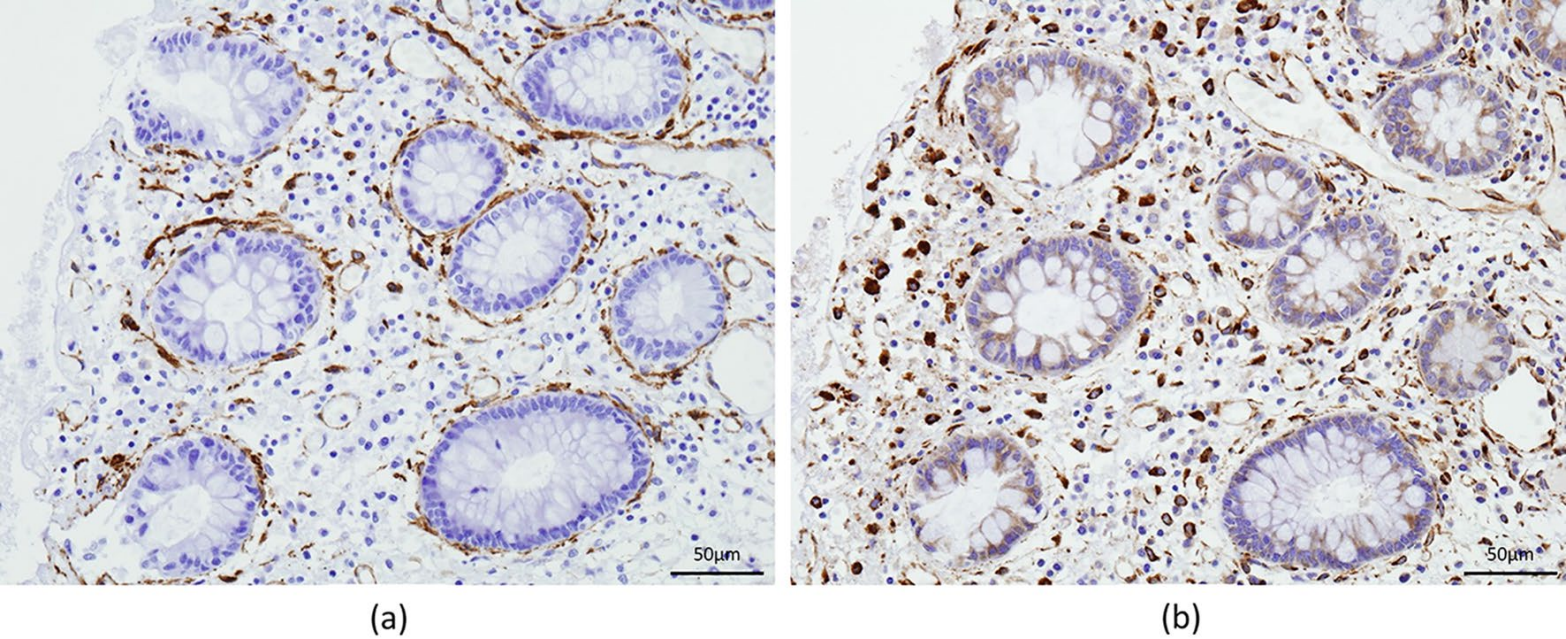
Immunostaining for α smooth muscle actin and HSP47. Representative immunohistochemical staining images for HSP47 and smooth muscle actin in 3-µm-thick serial sections of intestinal biopsy tissues obtained from patients with Crohn’s disease.

Immunohistochemical studies were performed on specimens biopsied from the colonic mucosa of patients with CD to confirm HSP47 localization in these tissues. The expression of collagenous tissue was confirmed by assessing α-smooth muscle actin (α-SMA) expression, which colocalized with HSP47 (Fig. 6).

### Effect of HSP47 inhibitor on human fibroblasts

**Figure 7:**
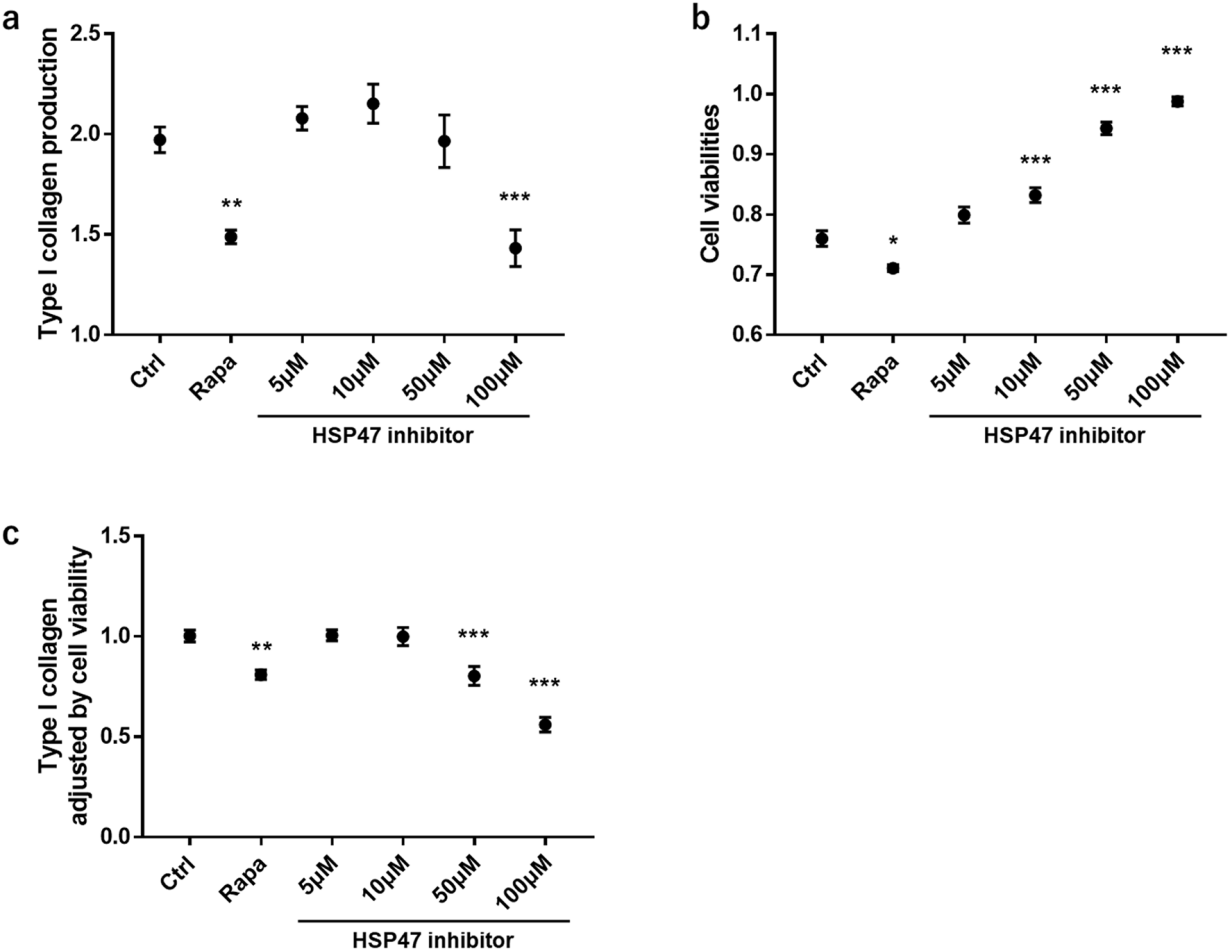
Effect of HSP47 inhibitor on collagen production. Normal human fibroblast cells treated with control, rapamycin (10 nM), or different concentrations of HSP47 inhibitors. (**a**) Type I collagen production in human fibroblast cells after 24 h of treatment. (**b**) Cell viabilities of human fibroblasts after treatment. (**c**) Type I collagen production adjusted by cell viabilities. Rapa, rapamycin; HSP, heat shock protein. *P < 0.05; **P < 0.01; ***P < 0.001 (one-way analysis of variance test with a post-hoc Dunn’s test).

We investigated the effect of HSP47 inhibition on fibrosis prevention in human fibroblasts. The HSP47 inhibitor (100 μM) significantly reduced type I collagen production (Fig. 7a) without decreasing cell viability (Fig. 7b). An mTOR inhibitor, rapamycin, was used as a control and also suppressed type I collagen production; however, unlike the HSP47 inhibitor, rapamycin reduced cell viability (Fig. 6a,b). Type I collagen production normalized by cell viability decreased significantly after HSP47 inhibition in a dose-dependent manner (Fig. 7c). These results suggest that HSP47 inhibition exerts a protective effect against fibrosis without affecting cell viability.

## Discussion

In the present study, we demonstrated that serum HSP47 and anti-HSP47 antibody levels were significantly higher in patients with CD than in patients with UC. Serum anti-HSP47 antibody levels were found to reflect CD activity. Moreover, serum anti-HSP47 antibody levels were significantly higher in patients with CD with intestinal stricturing than in patients without it. In addition, HSP47 colocalized with α-SMA, a marker for myofibroblast that plays a central role in the pathogenesis of fibrosis, suggesting its relevance in fibrosis in CD.

Intestinal stricturing is one of the major complications of CD and is often recurrent despite surgical and immunosuppressive therapies. More than one-third of patients with CD show a stenosing phenotype^13^. Inflammation of intestinal mucosa is associated with infiltration by immune cells, leading to epithelial damage. Deposition of collagen in the extracellular matrix during the wound-healing cycle causes fibrotic stricture^8^. Although the pathogenesis of inflammation in IBD, including CD, has been extensively investigated, the precise mechanisms underlying the development of fibrotic stricture in CD are not completely understood. In addition, intestinal stenosis in CD can be observed in the absence of luminal inflammatory symptoms^13^. Therefore, identification of a target that participates in collagen production and deposition is required.

HSP47 is a molecular chaperone exclusively expressed in the ER and is involved in the processing, assembly, folding, and secretion of collagens^11,14^. During fibrosis, collagen secretion is induced. HSP47 can be detected around fibrotic lesions^15,16^. An in vitro study using human lung fibroblast cells revealed that HSP47 is induced by a profibrotic mediator, tissue growth factor-β^14^. In an experimental study involving a murine model of induced liver cirrhosis, HSP47 expression was positively correlated with the degree of fibrosis^12^. Furthermore, serum HSP47 and anti-HSP47 levels are elevated in patients with connective tissue diseases^17^. The role of HSP47 in intestinal fibrosis has been investigated by Kitamura et al., who found that transcription of *SERPINH1*, which encodes HSP47, was elevated in the colon tissue of mice with spontaneous colitis, and that HSP47 was distributed throughout the collagenous tissues^18^. Increased HSP47 expression has also been observed in the intestinal tissues of patients with CD^19^.

In this study, we observed that serum HSP47 levels and anti-HSP47 antibody levels were significantly higher in patients with CD than in patients with UC. ROC curve analysis revealed that both HSP47 antigen and antibody levels can be used as serological markers for differentiating between CD and UC. Our observation is similar to that of a previous study, in which high HSP47 levels were detected in the sera of patients with CD^20^. However, this previous study did not observe a positive correlation with CD activity. We also investigated the differences in the expression of serological markers in patients with active or inactive CD and found that only anti-HSP47 levels, but not HSP47 levels, were high in patients with active CD. We compared HSP47 levels between CD patients with and without intestinal stricturing and found that HSP47 levels tended to be high in patients with intestinal stricturing. We did not observe any significant difference in serum HSP47 levels between the healthy control group and the CD group^20^. The heterogeneity of the patient cohort in this study could explain why this result is inconsistent with a previous study. Indeed, heterogeneity of the study cohort has been shown to diminish the diagnostic ability of HSP47 for lung fibrosis^21^.

Research into the pathophysiology of IBD has revealed that adipose tissue-derived free fatty acids and adipocytokines are involved in inflammation in IBD^22,23^. Another study suggested that HSP47 is associated with fibrosis of adipose tissue^24^. In this study, we observed colocalization of HSP47 with α-SMA in intestinal tissue from patients with CD. As described previously, HSP47 is considered a candidate for the anti-fibrotic treatment of CD^25^. Indeed, HSP47 inhibition has been shown to hinder the profibrotic mechanism of lung fibroblasts^26^. In this study, we observed that HSP47 inhibition significantly suppressed collagen production in fibroblasts. The mTOR inhibitor, rapamycin, also reduced collagen production, but this was accompanied by increased cell death. Since the HSP47 inhibitor inhibited collagen production without affecting cell viability, it could be an ideal agent for fibrosis prevention. Further investigation of the effect of HSP47 inhibitor on intestinal tissues from CD patients is desired to confirm the mechanistic role of HSP47 in the formation of fibrosis in CD.

Our study has certain limitations. First, this study aimed to identify noninvasive surrogate markers of CD activity—particularly fibrotic stenotic lesions—that can be isolated from blood. Further studies using intestinal biopsy samples from patients with CD are required to investigate the association between serum HSP47 levels and local HSP47 levels in the intestinal mucosa of patients with CD. Second, we demonstrated that HSP47, especially the anti-HSP47 antibody, can be used as a biomarker for CD and its activity. Although the molecular mechanism by which HSP47 contributes to collagen deposition remains to be elucidated, inhibition of HSP47 can constitute a novel antifibrotic therapy. Further research is required to elucidate the mechanism by which HSP47 contributes to intestinal fibrosis in CD. In conclusion, we show that HSP47 is a biomarker for CD and positively correlates with disease activity. Thus, HSP47 may be a potential target for the development of antifibrotic CD therapies.

## Materials and methods

### Study population

This observational study included 26 with UC and 32 patients with CD. All patients had already been diagnosed with IBD prior to recruitment in this study. Diagnoses of UC and CD were made based on conventional clinical, laboratory, endoscopic, histopathological, and radiological parameters^27^. Disease evolution time was defined as the duration from disease onset to the date of HSP47 measurement. For patients with UC, a clinical activity index (CAI) ≤ 4 was defined as being in remission and CAI > 4 was defined as being active stage^28^; for patients with CD, a Crohn’s disease activity index (CDAI) ≤ 150 was defined as being in remission and CDAI > 150 was defined as being active stage^29^. The Montreal Classification for IBD classifies CD subgroups according to factors such as age, inflammation distribution, and disease behavior^30^. The Montreal Classification categorizes age of onset as A1 for patients 16 years old or younger, A2 for patients 17–40 years old, and A3 for patients over 40 years old; the disease location as L1 for ileal, L2 for colonic, L3 for ileocolonic, and L4 for isolated upper disease; the disease behavior as B1 for nonstricturing and nonpenetrating, B2 for stricturing, and B3 for penetrating, further modified as p for cases involving perianal disease. The control group consisted of 22 randomly selected healthy individuals. We determined that a total number of 86 participants would provide the study with 80% power (P = 0.05; effect size: 0.46). The target number of participants was calculated using G*Power software (version 3.1.9.6; Germany)^31^ as in a previous report^20^. This study was conducted in accordance with the Declaration of Helsinki and approved by the Ethics Committee of Nagasaki University Hospital (approval numbers: 14052644, 13040149, and 11092637) and Tottori University Hospital (approval number: 1508A024). Written informed consent was obtained from all patients.

### Cell culture and treatment

Normal human fibroblasts (ACEL, Inc., Kanagawa, Japan) were used in this study. The cells were maintained in Dulbecco’s Modified Eagle Medium (DMEM) (Nacalai Tesque, Kyoto, Japan) supplemented with 10% fetal bovine serum (Invitrogen) and 1% penicillin–streptomycin (Nacalai Tesque). The cells were cultured in a humidified incubator at 37 °C in an atmosphere of 95% air and 5% carbon dioxide. When required, the cells were treated with DMEM containing 5, 10, 50, or 100 μM of HSP47 inhibitor (TECHNO SUZUTA, Nagasaki, Japan). An mTOR inhibitor, rapamycin, was used as a internal control. After 24 h incubation, cell viabilities were analyzed using the WST-8 Cell Proliferation Kit (Funakoshi, Tokyo, Japan), and type I collagen production was analyzed using an enzyme-linked immunosorbent assay (ELISA) kit (ACEL, Inc., Kanagawa, Japan).

### Measurement of serum HSP47 concentration and autoantibody titers

The serum concentration of HSP47 was measured using sandwich ELISA, as described previously^32^. The HSP47 autoantibody titers were measured using ELISA, as described previously^33^. In brief, recombinant HSP47 diluted to 1 μg/mL in 50 mM sodium carbonate buffer was immobilized on a 96-well microplate. After blocking with 2% bovine serum albumin and rinsing with phosphate-buffered saline, the wells were incubated with human sera (diluted 100-fold). Specific binding of serum IgG to HSP was detected by incubating with horseradish-peroxidase-conjugated antibodies specific to the γ-chain of human IgG (BioSource, Camarillo, CA, USA) and 3,3′,5,5′-tetramethylbenzidine solution. Antibody titer was determined by measuring the absorbance at 450 nm.

### Immunohistochemistry

Intestinal mucosa biopsy specimens were fixed with 10% formalin and embedded in paraffin. Three-micrometer-thick sections were deparaffinized and dehydrated, followed by antigen retrieval in ethylenediaminetetraacetic acid buffer (pH 9.0) at 95 °C for 30 min. The slides were then incubated with 3% hydrogen peroxide for 10 min. After incubation with blocking solutions and rinsing with Tris-buffered saline (TBS), the slides were incubated with anti-HSP47 antibody (1:15,000; M16.10A1; Enzo Life Sciences, Inc.) or anti-α-SMA (1:1000; Cosmo Bio Co., Ltd.) at 24 °C for 60 min. The slides were then rinsed with TBS and incubated with peroxidase-labeled corresponding secondary antibody for 30 min at room temperature. Peroxidase activity was detected using diaminobenzidine. Hematoxylin was used for counterstaining.

### Statistical analysis

Statistical analysis was performed using IBM SPSS 28.0.1 (IBM, Somers, NY, USA). Continuous variables were expressed as mean ± standard deviation or median with interquartile range, according to the distribution. The Kolmogorov–Smirnov test was used to assess normal distribution. Categorical variables were expressed as percentages. Differences between two groups were analyzed using Student’s *t* test for normally distributed variables, the Mann–Whitney *U* test for non-normally distributed variables, or a Chi-squared test for categorical variables. Continuous variables among the three groups were compared using one-way analysis of variance for normally distributed variables or the Kruskal–Wallis test for non-normally distributed variables. For significant differences, the Scheffe test was used for post-hoc analysis of normally distributed variables or the Dann–Bonferroni test for non-normally distributed variables. ROC curve analysis was performed to determine the optimal cut-off values of HSP47 and anti-HSP47 antibody levels for differentiating between CD and UC. Furthermore ROC curve analysis was performed to determine the optimal cut-off values of HSP47 and anti-HSP47 antibody levels for differentiating CD with or without stricturing. Optimal cut-off values were determined by minimizing the square of the distance between a point (sensitivity of 1, 1-specificity of 0) and any point on the ROC curve. Two-tailed P values < 0.05 were considered statistically significant.

## Data Availability

This study's datasets are available upon reasonable request.
